# Numerical Simulation of Dry Ice Compaction Process: Comparison of Drucker-Prager/Cap and Cam Clay Models with Experimental Results

**DOI:** 10.3390/ma15165771

**Published:** 2022-08-21

**Authors:** Maciej Berdychowski, Jan Górecki, Aleksandra Biszczanik, Krzysztof Wałęsa

**Affiliations:** Faculty of Mechanical Engineering, Institute of Machine Design, Poznan University of Technology, 60-965 Poznań, Poland

**Keywords:** Drucker-Prager/Cap, cam clay, compaction, densification, dry ice, carbon dioxide

## Abstract

This article presents the results of a numerical experimental study on the simulation of the dry ice compaction process. The first part of the article presents a description of the material used, material models and the methodology of experimental research. In the second part, numerical and experimental study results are presented. For the purpose of comparison, a parametric method based on the residual sum of squares was used. The application of the indicated method fills the gap in the available literature as the authors are not aware of any existing data from previous studies on the method of comparing the results of numerical tests in terms of the obtained results and the change of the value of the tested parameter as a function of another variable. The results of this study can be useful in research work aimed at further development of the process of extrusion and compaction of dry ice using Drucker-Prager/Cap and modified Cam-Clay material models for instance for optimization of geometric parameters of parts and components of the main assembly of the machine used in the process of dry ice extrusion.

## 1. Introduction

The current advancement of the simulation techniques used allows for increasingly more accurate estimation of working loads occurring in the processing of comminuted materials. This is especially important in their densification considered to be a very energy-intensive process [1].

The densification process is widely used e.g., in the pharmaceutical industry [2], in the production of biofuels [3] and in the processing of waste materials [4]. Particular attention must be paid to limiting the working load, especially when compacting waste materials. Steps taken to limit the indicated value result in direct reduction of energy, e.g., electrical energy [5], consumption. Their indirect effect involves a reduction in the emission of greenhouse gases to the atmosphere [6]. This is particularly important in the recycling and refining of these materials.

One of the materials undergoing densification is crystallized carbon dioxide. In the solid phase and under normal conditions, carbon dioxide has a temperature of −78.5 °C and it sublimates [7]. Hence, it is widely used, for example, in cooling processes [8,9] and cleaning of surfaces [10]. However, if it is to be used effectively it needs to be densified, which allows to reduce the material’s sublimation rate [11].

**Figure 1:**
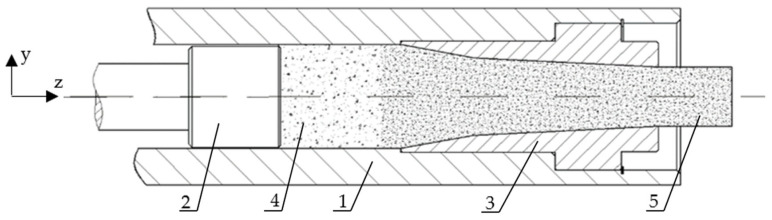
The main part of the piston-type pelletizer. 1—compaction chamber, 2—piston, 3—die, 4—dry ice before compression, 5—compressed dry ice.

The process can be carried out using commercially available equipment. The principle of its operation is based on piston technology [12], with an example drawing of a working unit shown in Figure 1.

At the initial stage of the machine cycle, the compaction chamber (Figure 1, label 1) is filled with comminuted material (Figure 1, label 4). In the next stage, the piston (Figure 1, label 2) is moved in order to reduce the capacity of the chamber and, as a result, the material is compressed. During material compaction in the above-described technique, we observe uniaxial compaction, where the material stress state σ can be described by the following tensor, Equation (1):

(1) $$\sigma =\left[\begin{array}{ccc} \sigma_{x} & 0 & 0\\ 0 & \sigma_{y} & 0\\ 0 & 0 & \sigma_{z} \end{array}\right],$$

The value of stress increases as a function of the piston travel distance (displacement). It is growing until the values of the force on the piston and the resistance to motion of the material in the working chamber and the die become equal (Figure 1, label 3). When they have become equal, the compaction stage is completed and the extruding stage begins, during which the final shape is given to the compacted material.

The article deals with the topic of comparing the results of the numerical analysis of the densification process with the empirical results. Diarra et al. 2012 pointed out that the Discrete Element Method (DEM) and the Finite Element Method (FEM) are used successfully in the compaction process simulation [13]. Harthong et al. 2009, specified that the DEM are used successfully in compaction of loose materials up to zero relative density 0.8 [14]. Above this value, he suggests using the Meshed Discrete Element Method (MDEM) and the FEM. Support for the relevance of the use of FEM methods above a relative density of 0.8 can also be found in other literature sources, such as Brewin et al. 2008 and Wilczyński et al. 2021 [15,16].

Due to sublimation, dry ice is compacted to a relative density value above 0.8. For this reason, the authors decided to apply the FEM analysis in the article. Two mathematical models have been described in the literature that allow to simulate compaction of loose materials, i.e., the Drucker Prager-Cap (DPC) [17] and modified Cam-Clay (CC), [18,19] models.

In the second section of the article the authors describe the models used and set the parameters for each of them. The methodology for performing the empirical study is also presented. In the third section of the article the research results are presented in the form of densification characteristics obtained in experimental work and by way of computer simulations. The presented results made it possible to assess which model offers a better representation of the process performed under laboratory conditions.

The authors of the article have performed an extensive literature review but have not come across any study comparing the results of numerical simulations performed using the DPC and MCC models. The novelty of the article is to compare the results of the analysis with the results of empirical studies and to try and determine which model allows for better representation of the results of empirical studies.

## 2. Materials and Methods

### 2.1. Materials

#### 2.1.1. Powder

Comminuted dry ice was obtained by expanding liquid carbon dioxide at a temperature of −18 °C, kept in a tight container under a pressure of 20 bar. To obtain the material in a solid state, liquid CO_2_ was rapidly adiabatically expanded to atmospheric pressure. This resulted in the crystallization of the material. The density of dry ice in the powdered form is 550 kg/m^3^ [20].

For the purpose of empirical testing, the comminuted material was stored in foamed polystyrene containers. The use of an insulated container reduced the rate of sublimation. Additionally, the containers were used to cool the test stand components in order to reduce their temperature down to a level close to the temperature of the test material.

#### 2.1.2. Compaction

In the tests the cylindrical pellets were compressed in a closed chamber using the piston technology. For empirical work the authors used a test stand of own design dedicated to the compression of dry ice specimens in the piston technology between the grips of the MTS Insight 50 kN (MTS Systems Corporation, Eden Prairie, MN, USA) universal testing machine. A detailed description of the experimental setup was provided in previous studies [21].

The tests involved measuring the value of the force *F_Z_* imparted to the compacting piston with a 30 mm diameter. The measurement was performed, using an accuracy class 0.01, 50 kN strain gauge sensor of the MTS Insight 50 kN system.

During empirical testing, the material was compacted to the assumed specimen strain value. This made it possible to obtain specimens with density limits values of approx. 1650 kg/m^3^.

#### 2.1.3. Dry Ice Elastoplastic Properties in the Function of Density

During compaction, the structure of the material changes. In the initial phase of the compaction process loose material acquires a porous structure. The shape of the specimen depends on the geometric parameters of the compaction chamber and the material has a low Young’s modulus E and Poison’s ratio values. During compaction the pore space between the material’s particles is reduced. As a result, the values of parameters *E* and *ν* increase.

Biszczanik et al., 2021 and 2022, reported on the results of experimental tests of the effect of the value *ρ* on the value of the Poison’s ratio *ν* and modulus *E*. Based on the obtained characteristics, the following equations were formulated [21,22]:

(2) E(ρ)=1.328ρ−1282.93,

(3) $$\nu \left(\rho \right)=-7.1226\times 10^{-2}+\frac{0.58544\;\rho^{8.223076}}{1220.08^{8.223076}+\rho^{8.22308}}.$$

these equations were used in numerical simulations to describe the change in material behavior in the elastic deformation region.

### 2.2. Material Models

The numerical tests described in this article were performed using Abaqus/Explicit 2020 software from Dassault Systémes. This environment allows for numerical analysis using both discussed models.

#### 2.2.1. The Drucker-Prager Cap (DPC) Model

**Figure 2:**
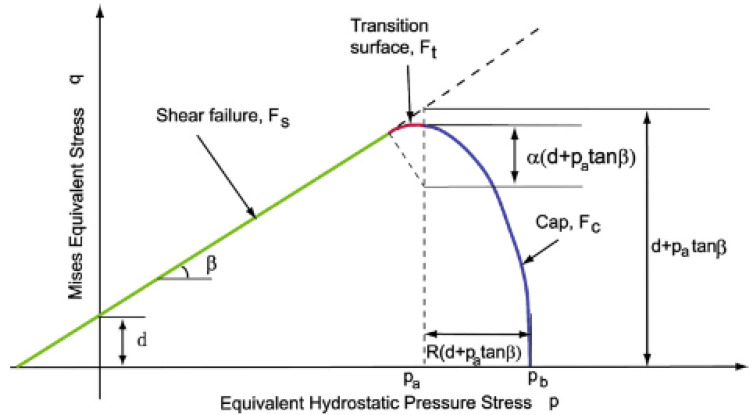
Drucker-Prager Cap model: yield surface in the *p*–*q* plane [23].

As indicated at the beginning of this article, one of the models used for the FEM analysis of the dry ice compaction process was the DPC model. The DPC model falls into the group of phenomenological elastic-plastic models. The DPC consists of three failure surfaces described on the *p*-*q* surface (*p* is the hydrostatic stress, *q* is the Mises equivalent stress), which was presented in Figure 2.

The model contains Drucker-Prager failure line defined as *F_S_*, Cap surface *F_C_* and transition surface *F_t_*. Diarra et al. 2012, indicated that the equations describing the model surfaces can be expressed in the form shown below [13]:

(4) $$F_{S}=q-p\;tan\beta -d=0,$$

(5) $$F_{C}=\sqrt{{\left(p-p_{a}\right)}^{2}+{\left(\frac{Rq}{1+\alpha -\alpha /cos\beta }\right)}^{2}}-R\left(d+p_{a}tan\beta \right)=0,$$

(6) $$F_{t}=\sqrt[{}]{{\left(p-p_{a}\right)}^{2}+{\left[q-\left(1-\frac{\alpha }{cos\beta }\right)\left(d+p_{a}tan\beta \right)\right]}^{2}}-\alpha \left(d+p_{a}tan\beta \right)=0,$$

where *β* is friction angle, *R* is the eccentricity, *d* powder cohesion, *p_a_* is an evolution parameter. The α parameter ensures smooth transition between the Cap surface and the shear failure segment.

Since, the model has been extensively described in the literature of the subject, in this article, its description has been limited to the final introduction of equations describing the change in parameter values as a function of material density and for more details reader can refer to the referenced articles [13,17].

The equation describing a change in the value of *F_S_* requires β and *d* to be defined. Talaśka 2018, determined an equation in experimental studies, that describes a change in the value of dry ice powder cohesion *d* as a function of material’s relative density $\rho_{w}$ [24]. He expressed the results of these studies in the following equation:

(7) $$d\left(\rho_{w}\right)=354.59\rho_{w}{}^{4}+1250.5\rho_{w}{}^{3}-1648.1\rho_{w}{}^{2}+964.48\rho_{W}-208.862.$$

after transforming Equation (7), it was possible to describe the change in *d* as a function of density *ρ*.

(8) $$d\left(\rho \right)=-5\times 10^{-11}\rho^{4}+3\times 10^{-7}\rho^{3}-6\times 10^{-4}\rho^{2}+0.5935\rho -208.862.$$

Talaśka 2018, presented also the results of experimental studies describing a change in compaction stress $\sigma_{Z}$ as a function of $\rho_{w}$. He described the results of these studies by means of the following equation [24]:

(9) $$\sigma_{Z}\left(\rho_{w}\right)=-332.48\rho_{w}{}^{4}+1185.8\rho_{w}{}^{3}-1580.7\rho_{w}{}^{2}+969.39\rho_{W}-205.01.$$

after transforming Equation (9), it was possible to describe the change in $\sigma_{Z}$ as a function of *ρ*.

(10) $$\sigma_{Z}\left(\rho \right)=-5\times 10^{-11}\rho^{4}+3\times 10^{-7}\rho^{3}-6\times 10^{-4}\rho^{2}+0.5762\rho -205.01.$$

Moreover, Han et al. 2008, presented an equation, which allows to determine the value β from values $\sigma_{Z}$ and *d*. On the basis of Equations (8) and (10) the following equation can be written down, from which the value β as a function of ρ can be determined [17]:

(11) $$\beta \left(\rho \right)=tan^{-1}\left[\frac{3(\sigma_{Z}\left(\rho \right)+d\left(\rho \right)}{\sigma_{Z}\left(\rho \right)}\right].$$

for the equation describing the change in *F_C_* parameters *R* and *Pa* must be defined. However, Zhang et al. 2010, in their studies indicate that values of additional parameters, $p_{A}$ and $q_{A}$, must also be determined. They can be calculated using Equations (12) and (13) [25,26]. Both these equations require the use of stress values $\sigma_{Z}$ and $\sigma_{x}$. They were determined based on the published results of studies by Biszczanik et al. 2021 [21].

(12) $$p_{A}=-\frac{1}{3}\sigma_{z}\left(1+2\frac{\sigma_{x}}{\sigma_{z}}\right)$$

(13) $$q_{A}=\frac{\sigma_{z}}{\sqrt[{}]{2}}\left(1-\frac{\sigma_{x}}{\sigma_{z}}\right).$$

Parametr *Pa* was described by the following quadratic equation, by Diarra et al. 2012 and Han et al. 2008 [13,17].

(14) $$A\cdot P_{a}{}^{2}+B\cdot P_{a}+C=0,$$

where

(15) $$A=2\cdot tan^{2}\beta .$$

(16) $$B=3q_{A}+4d\cdot tan\beta .$$

(17) $$C=2d^{2}-3p_{A}q_{A}-2q_{A}{}^{2}.$$

after transforming Equation (14) to determine its zero, an equation describing the value *Pa* was determined.

(18) $$P_{a}=\left(-B+\sqrt{\Delta }\right)/2A.$$

Brewin et al., 2008, formulated Equations (17) and (18) to determine the values of parameters *R* and *P_b_*, based on the previously determined values $P_{a}$, $p_{A}$ and $q_{A}$ [15].

(19) $$R=\sqrt{\frac{2}{3}}\frac{P_{a}-p_{A}}{q_{A}}.$$

(20) $$p_{b}=p_{A}+R\left(p_{A}\cdot tan\beta +d\right).$$

The presented mathematical relationships and empirical study results allowed to determine the value of DPC model parameters for the determined values of dry ice density. The results are given in the following Table 1.

#### 2.2.2. The modified Cam Clay (MCC) Model

As indicated at the beginning of the article, the other model used to simulate the compaction of crystallized carbon dioxide was the Modified Cam-Clay plasticity model (MCC), which was originally developed to describe the behavior of non-cohesive soils, such as saturated clays. Cam-Clay plasticity model describes the inelastic behavior of a material using the yield function that depends on three stress invariants, the law of plastic flow to determine the rate of plastic deformation, and the theory of plastic hardening, which has an effect on the size of the yield surface depending on the inelastic volumetric strain [23]. The model itself has been used frequently by numerous researchers [15,18,19] to simulate the compaction of materials, also materials other than soils.

The Cam-Clay Model implemented into Abaqus is based on the yield surface:

(21) $$f\left(p,q,r\right)=\frac{1}{\beta^{2}}{\left(\frac{p}{a}-1\right)}^{2}+{\left(\frac{t}{Ma}\right)}^{2}-1=0,$$

where

(22) $$p=-\frac{1}{3}trace\left(\sigma \right)—\mathrm{is}\;\mathrm{the}\;\mathrm{equivalent}\;\mathrm{pressure}\;\mathrm{stress}$$

(23) $$q=\sqrt{\frac{3}{2}\;S:S},—\mathrm{is}\;\mathrm{the}\;\mathrm{Mises}\;\mathrm{equivalent}\;\mathrm{stress}$$

(24) $$r^{3}=\frac{9}{2}S:S\cdot S—\mathrm{is}\;\mathrm{the}\;\mathrm{third}\;\mathrm{stress}\;\mathrm{invariant}$$

(25) $$t=\frac{1}{2}q\left[1+\frac{1}{K}-\left(1-\frac{1}{K}\right){\left(\frac{r}{q}\right)}^{3}\right]—\mathrm{is}\;a\;\mathrm{deviatoric}\;\mathrm{stress}\;\mathrm{measure}$$

The MCC model is a phenomenological elasto-plastic model with hardening. Depending on the manner of defining the material hardening, which is closely related to the type of the Abaqus module (Standard or Explicit) used in the analysis, the system requires different parameters describing the material to be provided. In the analyses described in this article the authors used Abaqus Explicit, hence the HARDENING option was set for the TABULAR value. With settings defined in this way, the program requires entering the stress ratio at critical state, which is a constant that defines the slope of the critical state line, the initial volumetric plastic strain, Wet Yld Surf Size is a user-specified constant that can be a function of temperature or other variables, defines the size of the yield surface and is used to modify the shape of the yield surface. Next, the Flow Stress Ratio (K) is the ratio of the flow stress in triaxial tension to the flow stress in triaxial compression that determines the shape of the yield surface in the plane of the principal deviatoric stresses. Abaqus requires that the value of K lies between 0.778 ≤ K ≤ 1 to ensure that the yield surface remains convex.

Other material data such as initial density, Young’s modulus, Poisson’s ratio and volumetric plastic strain were defined identically in both models used. The values of all material parameters are given in Table 2.

#### 2.2.3. Methodology of Experimental Test

The objective of the study was to empirically characterize the change in compaction force as a function of compaction piston travel distance *s*, in order to allow validation of selected numerical models of the compaction process. To achieve this, the authors used a method described in the literature consisting in determining compaction curves with material stress relaxation [21]. To this end, dry ice was compacted in a specially designed stand, and then the force was released, and the free motion of the system was allowed. During the test, the values of the force exerted by the piston and its travel distance were recorded with the software dedicated to the testing machine used for the tests.

**Figure 3:**
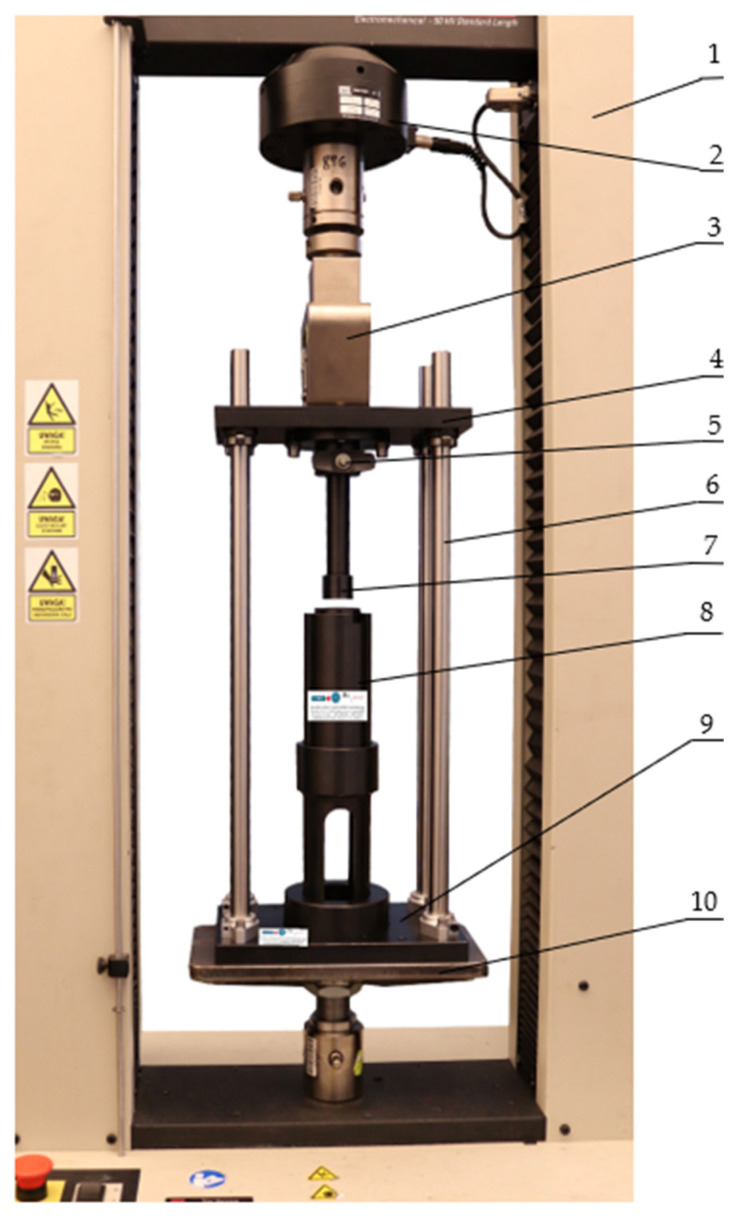
The stand for the testing of the elasticity modulus of dry ice as a function of density: 1. The MTS machine, 2. Machine sensor, 3. Machine grip, 4. Upper plate, 5. Pin, 6. Guide assembly, 7. Piston, 8. Compacting sleeve assembly, 9. Lower plate, 10. Machine base.

The tests were carried out using the MTS Insight universal tester with 50 kN force capacity (MTS Systems Corporation, Eden Prairie, MN, USA (MTS Systems GmbH, Berlin, Germany)) equipped with a strain gauge sensor for force measurement and a displacement transducer, both 0.5 accuracy class. The machine was controlled by a dedicated Test Works 4 software (MTS Systems Corporation, Eden Prairie, MN, USA). A test stand for empirical verification of the compaction stress value during the dry ice agglomeration process was installed between the machine’s grips. This test stand was also used in the determination of the Young’s modulus of dry ice, where the tests and their results are reported in the publication [21]. The set of devices shown in Figure 3 were used to carry out the tests, during which it was possible to record the value of the force applied to the compacting piston as a function of its displacement. The output signals from the sensors of the MTS machine were transmitted to the HBM Spider 8 measuring amplifier (Hottinger Baldwin Messtechnik GmbH (HBM), Darmstadt, Germany), from which they were sent HBM’s Catman Easy program (Hottinger Baldwin Messtechnik GmbH (HBM), Darmstadt, Germany), version 3.5, compatible with the measuring amplifier. The acquisition and processing of the measurement signal values was performed with a frequency of 100 Hz. The ACN 220 (AXIS, Gdańsk, Poland) analytical balances for the hydrostatic measurement of density, with a measurement accuracy of up to 0.001 g was used to measure the mass of the input material and the final density of the specimen.

**Figure 4:**
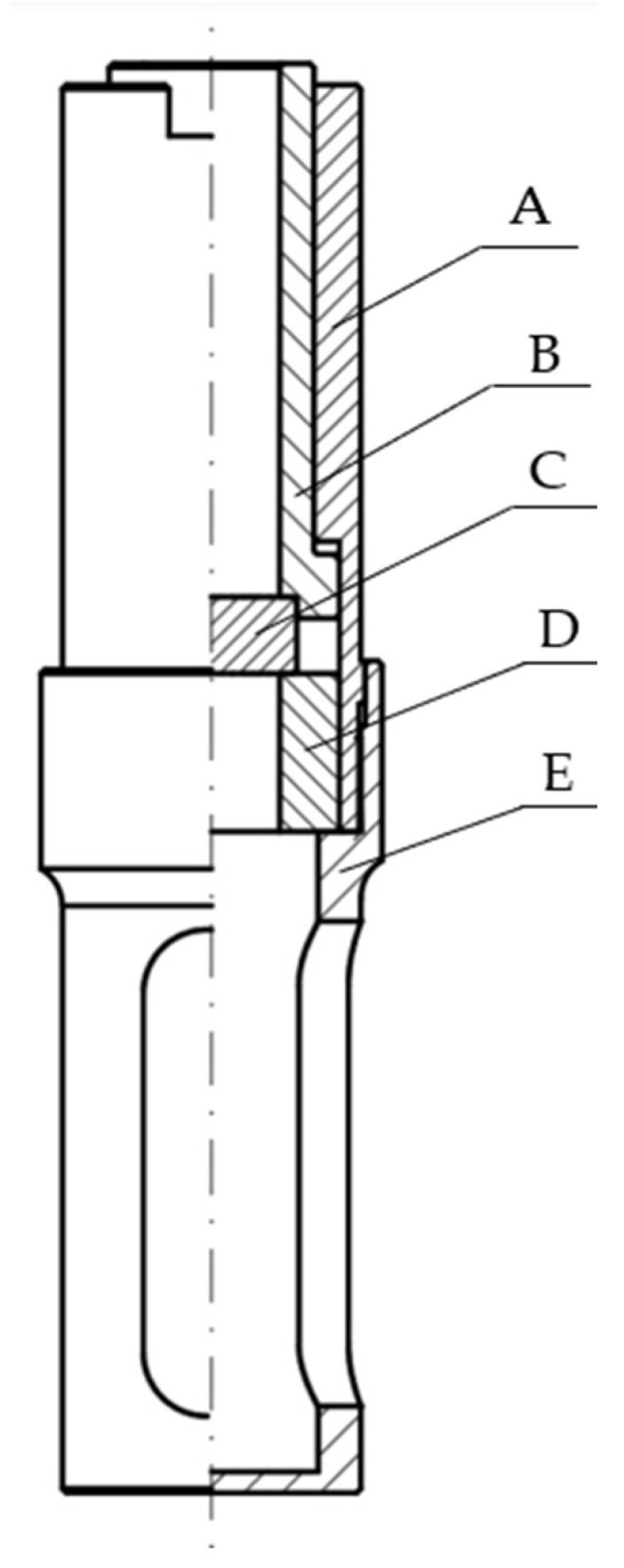
Compacting sleeve assembly, cross-section view: A. Upper sleeve, B. Compacting chamber, C. Compacting chamber bottom, D. Spacer sleeve, E. Lower sleeve [21].

The interior of the compacting sleeve assembly is illustrated in Figure 4.

At the first stage of the test, the test stand was set up on the testing machine to carry out the initial set-up, which consisted in aligning and checking the concentricity of the moving parts of the system, especially of the piston (Figure 3, label 7) in relation to the compacting chamber (Figure 4, label B), in which dry ice is compressed. The correctness of the positioning of the stand on the testing machine was checked by moving the piston into the chamber while monitoring the force value, and thus checking for additional resistance to movement. Once the stand was set up properly, the compacting sleeve assembly (Figure 3, label 8) was removed from it by pulling it out of the stand base (Figure 3, label 9) and the piston (Figure 3, label 7) was taken out by removing the locking pin (Figure 3, label 5) and sliding the piston out of the upper plate (Figure 3, label 4). The dismantled components were placed in a container with dry ice for about 30 min to cool down. Before starting the tests, the ambient temperature in the room was lowered to 18 °C and the humidity value did not exceed 50%. The test bench was cooled in dry ice to reduce its temperature to the temperature of the test material. The check measurements were taken with a type K thermocouple sensor and with a Testo 440 measuring instrument (Testo, Pruszków, Poland, which indicated the temperature of the test stand components of ca. −65 °C, however after the test stand was removed from the dry ice the temperature level was gradually rising due to the positive ambient temperature. On completion of the test cycle the temperature in the compaction chamber fell within the range of –40 to –45 °C, which was still sufficient to reduce material sublimation. Under industrial conditions, the pelletizers operate in industrial buildings or rooms which must have very good ventilation, but where no specific ambient conditions are required, so it was decided that the tests would also be carried out in a laboratory room and not under the reduced ambient temperature conditions for the system. On the lapse of the preliminary cooling time, the test stand was reassembled on the machine and the referencing of the piston surface in relation to the compaction chamber was performed, after which the stand was subjected to cooling for further 5 min. After this time, a predetermined amount of dry ice was poured into the compaction chamber located inside the compaction sleeve assembly. The amount depended on the expected density of the test sample after compaction. In the next step, the piston, together with the compaction sleeve system, was mounted back on the part of the system that remained on the testing machine and the measurement started, according to the algorithm programmed in Test Works 4. At the same time the recording of the results in the Catman Easy program was turned on. The machine program performed a sequence of work in the same order as described in study [21] when determining the Young’s modulus value of dry ice, i.e.,

1. The upper grip of the machine together with the piston arrived at the reference position with the initial velocity.
2. Initial downward motion was performed (with 1 mm/s travel speed) until a resistance force value of 50 N was detected.
3. The test was initiated. The machine grip moved downwards together with the mounted upper plate and the piston at a test speed of 5 mm/s until the specimen height of 24 ± 0.05 mm was reached.
4. The assembly retracted with a speed of 5 mm/s until a force value of 0 N was obtained.
5. The piston retracted with the final test speed to a height approximately 60 mm above the reference position to facilitate the removal of the sleeve and specimen for measurement.

On completion of the test, a resulting cylindrical specimen of the compressed dry ice was taken out of the compaction chamber and the density of the sample was measured using an analytical balance with a hydrostatic weighing kit. The stand components were again removed and placed for cooling in dry ice for another 5 min, and then the next compression test was performed.

The tests were repeated ten (10) times to yield the curve giving the average values of compaction force *F_Z_* determined for *s* at 0.2 mm intervals

#### 2.2.4. Compaction Simulation Model

**Figure 5:**
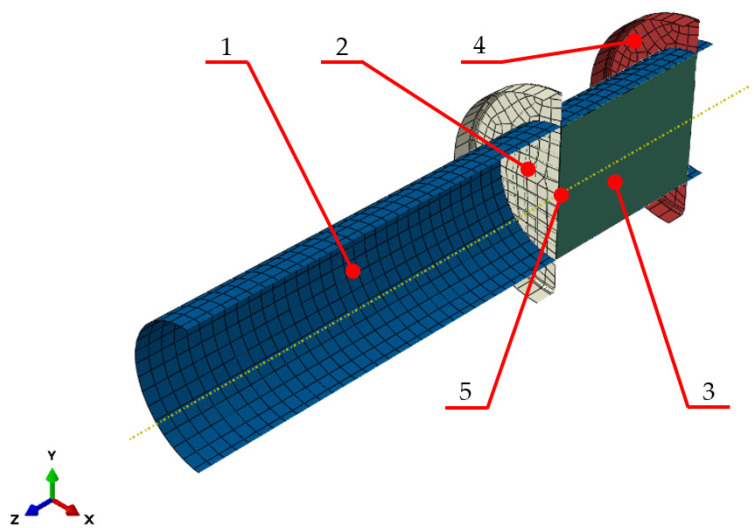
Numerical model: 1 sleeve, 2 punch, 3 compacted dry ice, 4 bottom closing disc, 5 compaction force measuring point.

The prepared numerical model reflects the compaction process carried out using the punch-sleeve assembly with a 30 mm diameter, which allows the results of the numerical calculations to be verified against the experimental results. The numerical model used for the calculations is shown in Figure 5. It is composed of 4 parts: the compacted material (Figure 5, label 3), forming sleeve (Figure 5, label 1), bottom closing disc (Figure 5, label 4) and top closing disc acting as the compacting piston (Figure 5, label 2). The compacted material was the only element modelled as a deformable object. It was illustrated as a cylinder with a diameter *D_C_* of 30 mm and the height *h_C_* of 39.95 mm. Other modelled elements with a function of a sleeve (Figure 5, label 1) in which the compaction process is taking place, were modelled as a discrete rigid part. A discrete rigid part is assumed to be rigid and is used in contact analyses to model bodies that cannot deform. The compacting piston (Figure 5, label 2) was modelled in an identical manner as the cylinder (Figure 5, label 1). In the model it is represented by a flat disc. The same geometry was also used to represent the base of the sleeve (Figure 5, label 4), in which the compaction takes place.

The sleeve and the bottom disc were modelled as non-deformable bodies and all of their degrees of freedom were removed. The compaction punch was allowed to have one degree of freedom in the form of a possibility to perform linear motion along the Z axis. During the simulation, the punch was moving at a speed of 5 mm/s along the Z axis.

A surface-to-surface contact interaction was defined in the model between the compacted material and the sleeve. A total of three interactions of this type were defined: between the material and the interior surface of the sleeve, between the material and the surface of the punch and between the material and the bottom disc closing the sleeve end. A friction coefficient value of µ = 0.1 was defined in the contact properties [24]. In the next step, the compacted material was ascribed with the parameters determined based on the experimental tests reported by Biszczanik et al. 2021 and 2022 [21,22]. The results described in this chapter, indicate, as mentioned previously, that the properties of the test material change along with the increasing degree of its compaction. To achieve the greatest possible convergence between the simulation and experimental results, these changes were mapped using the Abaqus USDFLD subroutine, in which the Yield Stress values (Table 1 and Table 2) were defined, as a criterion determining a change in material properties. This means that the input values of the parameters describing the material properties that Abaqus will acquire for a given calculation step will depend on the determined PEEQ (equivalent plastic stress) values obtained in the previous calculation step.

In the simulation model, a measurement point was defined on the inner surface of the compaction punch at its symmetry axis. It was used to record and read out the calculated values of the reaction force on the punch during the compaction process and to measure the displacement values. This way of defining the measurement point best reflected the force and displacement measurements made during the experimental tests.

According to results available in the literature, crystallized carbon dioxide with a density below 1000 kg/m^3^ does not have a cohesive form and the values of its mechanical parameters such as *E* and *ν* are close to zero. For this reason, the numerical analyses fell within the range of densities starting from 1050 kg/m^3^.

Harthong et al. 2009, specified that the FEM methods can be used successfully in the simulation of compaction of materials from zero relative density [14]. Additionally, based on information in the literature, the value of mechanical parameters such as *E* and ν of dry ice with a density below 1000 kg/m^3^ is close to 0. Therefore, the simulations and further work related to the comparison of its results with the results of the experimental tests were carried out from a piston position at 85 mm, which corresponded to the material density of 1050 kg/m^3^.

## 3. Results

**Figure 6:**
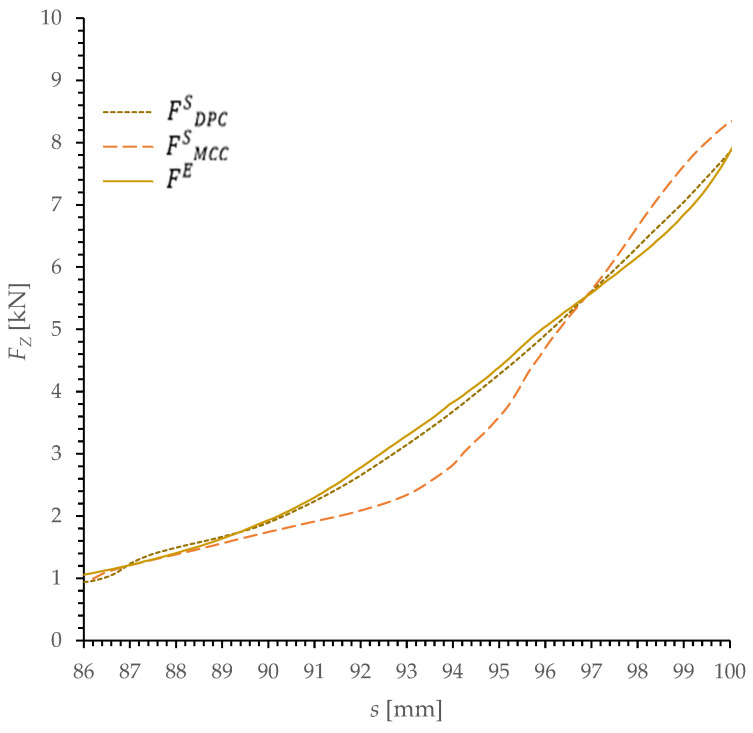
Characteristic of the change of value *F_Z_* as a function of *s* for the empirical test and the simulation results, F^S^_DPC_—results of numerical simulation using DPC model; F^S^_MCC_—results of numerical simulation using MCC model; F^E^—results of experimental research.

The results of the simulations and experimental tests of dry ice compaction process were represented as a characteristic of the change of force *F_Z_* as a function of displacement *s* in Figure 6.

**Figure 7:**
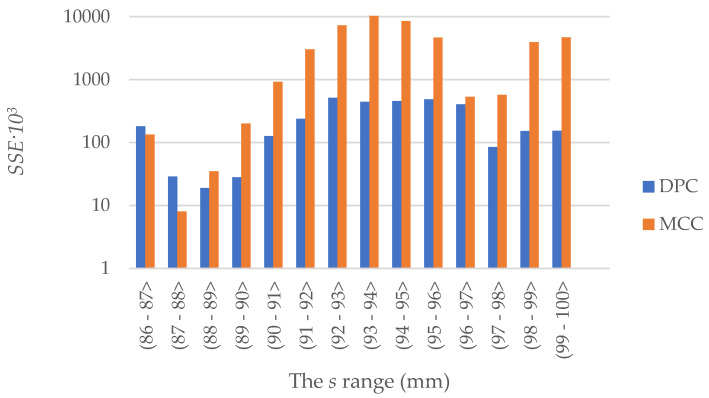
The change in the *SSE* value, in 1 mm intervals.

To assess the concurrency and quality of the representation of the experimental curve by the simulation results, the sum of squared errors of prediction (SSE) was calculated. The value of this measure is determined based on the sum of the squares of the difference in value between the simulation result *F^S^* and the experimental result *F^E^*, which can be written with the following equation:

(26) $$SSE=\sum_{s=86}^{100}{\left(F_{s}^{S}-F_{s}^{E}\right)}^{2}.$$

the *SSE* value was determined for both models in the *s* range from 86 to 100 mm, summing the value of the square of difference in 0.1 mm steps. Additionally, the *SSE* value was determined for *s* values in 1 mm intervals, which allowed an additional comparison of the quality of the empirical curve representation at 1 mm intervals of the CO_2_ compaction process. The results are presented in Table 3 and illustrated in Figure 7 in the form of a bar graph.

Maximum values of *F_z_* obtained during the simulation conducted using the DPC and MCC models are recorded in Table 4.

The percentage difference κ between a given value and the force limiting value $F_{Z}^{E}$ obtained during the empirical tests was additionally determined.

## 4. Conclusions and Results Discussion

The simulations conducted using the Drucker Prager-Cap (DPC) and Modified Cam-Clay (MCC) material models made it possible to obtain an approximate representation of the characteristic describing the change in the value of the compacting force *F_Z_* as a function of piston displacement *s.*

The graph reveals that for both models the value of the sum of squared errors of prediction *SSE* is not constant at individual sections of the *s.* At the initial stage of the simulation, i.e., for *s*-values between 86 and 87 mm, the results obtained using the two models are comparable. On the other hand, at the section between 87 and 88 mm the results of the simulation carried out using the MCC show a lower value of SSE compared to the results obtained from the DPC simulation. However, in subsequent intervals of value *s*, the value of the SSE for the simulation results obtained using the DPC was smaller or significantly smaller.

Over the entire test range, the SSE value is over 13 times higher for the results of the simulation carried out using the MCC than for the DPC results. However, the maximum *F_z_* values for both models are similar and do not differ significantly from the results obtained in empirical tests.

The above test results and their analyses allow to draw the following conclusions:

- both the MCC and the DPC model make it possible to determine an approximate *F_z_* value, where the value of the difference as compared to the experimental test result does not exceed 15%;
- the results presented indicate that the computer simulations of the dry ice compaction process using the DPC model offer a better representation of the curve describing the change in the force value during compaction.

The proposed method of comparing the results of the simulation tests obtained using two different models made it possible to find out which model better represents the change in the parameter under study. The study method described is similar to the methods known from the statistics literature [27,28]; however, the authors have not found any studies describing a similar or other methods allowing for a parametric comparison of the results of mechanical simulation tests. 

The results of this study could be applied to further research involving the following:

- Numerical simulation of the extrusion processes using the DPC and MCC material models, for the purpose of estimating the working load;
- Optimization of the geometric characteristics of the tools used in the compaction and extrusion of dry ice, to increase the process efficiency;
- Analysis of the energy consumption of the dry ice pelletization process with the use of a gravity roller press.

## Figures and Tables

**Table 1:** Values of DPC model parameters.

Material Cohesion [MPa]	Angle of Friction [deg]	Cap Eccentricity [Pa]	Init Yld Surf Pos [–]	Yield Stress [MPa]	Vol Plas Strain [–]	Young’s Modulus [MPa]	Poisson’s Ratio [–]
1.07	25.92	0.68	0.02	1.24	0	136.94	0.023
1.75	21.46	0.76	0.02	1.57	0.048	194.18	0.059
2.22	20.51	0.78	0.02	1.92	0.095	251.42	0.102
2.53	20.59	0.79	0.02	3.20	0.139	308.66	0.150
2.73	21.05	0.80	0.02	4.50	0.182	365.9	0.200
2.85	21.59	0.81	0.02	6.54	0.223	423.14	0.249
2.93	22.08	0.83	0.02	7.92	0.262	480.38	0.295
3.00	22.43	0.84	0.02	10.25	0.300	537.62	0.335
3.07	22.61	0.87	0.02	13.1	0.336	594.86	0.370
3.14	22.64	0.90	0.02	16.57	0.371	652.1	0.399
3.23	22.60	0.93	0.02	21.51	0.405	709.34	0.422
3.32	22.63	0.98	0.02	27.22	0.438	766.58	0.441
3.40	22.88	1.03	0.02	32.06	0.470	823.82	0.456

**Table 2:** Values of MCC model parameters.

Stress Ratio	Flow Stress Ratio	Wet Yld Surf Size	Init Vol Plas Strain [–]	Yield Stress [MPa]	Vol Plas Strain [–]	Young’s Modulus [MPa]	Poisson’s Ratio [–]
				1.24	0	136.94	0.023
				1.54	0.048	194.18	0.059
				2.09	0.095	251.42	0.102
				2.20	0.139	308.66	0.150
				2.63	0.182	365.9	0.200
				3.54	0.223	423.14	0.249
1	1	1	0.02	4.97	0.262	480.38	0.295
				5.96	0.300	537.62	0.335
				7.32	0.336	594.86	0.370
				8.64	0.371	652.1	0.399
				9.53	0.405	709.34	0.422
				9.99	0.438	766.58	0.441
				8.45	0.470	823.82	0.456

**Table 3:** *SSE* values.

Range of *s* Value [mm]	*SSE^DPC^*	*SSE^MCC^*
(86 –87〉	1.81 × 10^5^	1.34 × 10^5^
(87 –88	2.9 × 10^4^	8 × 10^3^
(88 –89〉	1.9 × 10^4^	3.5 × 10^4^
(89 –90〉	2.8 × 10^4^	2.01 × 10^5^
(90–91〉	1.27 × 10^5^	9.21 × 10^5^
(91 –92〉	2.38 × 10^5^	3.027 × 10^6^
(92 –93〉	5.16 × 10^5^	7.268 × 10^6^
(93 –94〉	4.42 × 10^5^	1.0334 × 10^7^
(94 –95〉	4.56 × 10^5^	8.474 × 10^6^
(95 –96〉	4.85 × 10^5^	4.660 × 10^6^
(96 –97〉	4.07 × 10^5^	5.31 × 10^5^
(97–98〉	8.5 × 10^5^	5.73 × 10^5^
(98 –99〉	1.53 × 10^5^	3.962 × 10^6^
(99 –100〉	1.54 × 10^5^	4.705 × 10^6^
**(86–100〉**	**3.32 × 10^6^**	**4.4833 × 10^7^**

**Table 4:** The maximum *F_z_* value.

	$!\max F_{Z}\;[\mathrm{kN}]$	κ[%]
$F_{Z}^{DPC}$	8.065	14.38
$F_{Z}^{MCC}$	8.328	11.59
$F_{Z}^{E}$	9.42	

## Data Availability

Not applicable.
